# ‘Whisper from the mitral valve’: delayed diagnosis of traumatic mitral valve regurgitation in an elderly farmer—a case report

**DOI:** 10.1093/ehjcr/ytaf399

**Published:** 2025-08-16

**Authors:** Shinnosuke Tsukamoto, Kensuke Matsumoto, Tomohiro Hayashi, Akitaka Yamada, Satoru Kawasaki

**Affiliations:** Division of Cardiovascular Medicine, Department of Internal Medicine, Hyogo Prefectural Tamba Medical Centre, 2002-7, Isou, Hikami-cho, Tamba, Hyogo 669-3495, Japan; Division of Cardiovascular Medicine, Department of Internal Medicine, Hyogo Prefectural Tamba Medical Centre, 2002-7, Isou, Hikami-cho, Tamba, Hyogo 669-3495, Japan; Division of Cardiovascular Medicine, Department of Internal Medicine, Hyogo Prefectural Tamba Medical Centre, 2002-7, Isou, Hikami-cho, Tamba, Hyogo 669-3495, Japan; Division of Cardiovascular Surgery, Department of Surgery, Kita-Harima Medical Centre, 926-250, Ichibacho, Ono, Hyogo 675-1392, Japan; Division of Cardiovascular Medicine, Department of Internal Medicine, Hyogo Prefectural Tamba Medical Centre, 2002-7, Isou, Hikami-cho, Tamba, Hyogo 669-3495, Japan

**Keywords:** Blunt chest trauma, Traumatic mitral valve injury, Mitral regurgitation, Case report

## Abstract

**Background:**

Traumatic mitral regurgitation (MR) is an exceptionally rare complication associated with blunt chest trauma, particularly following relatively low-impact injuries. In the critical and chaotic settings of polytrauma, its diagnosis is often delayed and can easily be overlooked. This oversight can lead to progressive haemodynamic deterioration and, ultimately, fatal outcomes.

**Case summary:**

A 73-year-old man sustained blunt chest trauma after falling ∼2.5 m from a truck bed. Initial assessment revealed a left haemopneumothorax and multiple rib fractures, with no audible heart murmur or signs of haemodynamic compromise. A chest drain was inserted, and he was discharged after conservative management. However, 3 weeks post-injury, a Grade III systolic regurgitant murmur was newly detected. Transthoracic and transoesophageal echocardiography revealed severe eccentric MR due to posterior mitral valve prolapse caused by chordal rupture. Intraoperative inspection confirmed a P2 chordal rupture with myxomatous degeneration. The patient underwent successful mitral valve repair and had an uneventful postoperative recovery.

**Discussion:**

This case highlights that traumatic MR, though extremely rare, can occur even after relatively minor trauma in elderly individuals, potentially due to underlying degenerative changes. Clinical signs of acute MR can be obscured by coexisting injuries, and auscultatory findings may only become apparent after these conditions have been treated. While echocardiography is crucial for identifying traumatic valvular dysfunction, its effectiveness can be limited by various factors associated with polytrauma. Therefore, a high index of suspicion is essential to prevent overlooking this potentially life-threatening complication in cases of blunt chest trauma.

Learning points

- Traumatic mitral regurgitation can occur following even less severe blunt chest trauma, particularly in elderly patients with preexisting myxomatous valve degeneration.
- Cardiac murmurs may be absent initially and appear later, especially after the resolution of coexisting conditions such as pneumothorax.
- A high index of suspicion must be maintained to avoid overlooking this potentially life-threatening complication in cases of blunt chest trauma.

## Introduction

Traumatic mitral regurgitation (MR) is an exceptionally rare complication of blunt chest trauma.^1–3^ In cases of polytrauma, multiple vital organ systems are often simultaneously affected, necessitating rapid assessment and prioritized life-saving interventions based on clinical urgency. Various comorbidities, such as central nervous system injuries, massive haemorrhage, respiratory failure, hypovolemia, and serious infections, may act alone or in combination to significantly compromise circulation. In such critical and chaotic settings, traumatic MR can be easily overlooked, potentially leading to progressive haemodynamic deterioration^4–6^ and ultimately death.^7^

Here, we report a rare case of traumatic MR diagnosed 3 weeks after the initial injury in an elderly patient who suffered blunt chest trauma from an accidental fall.

## Summary figure

**Figure:**
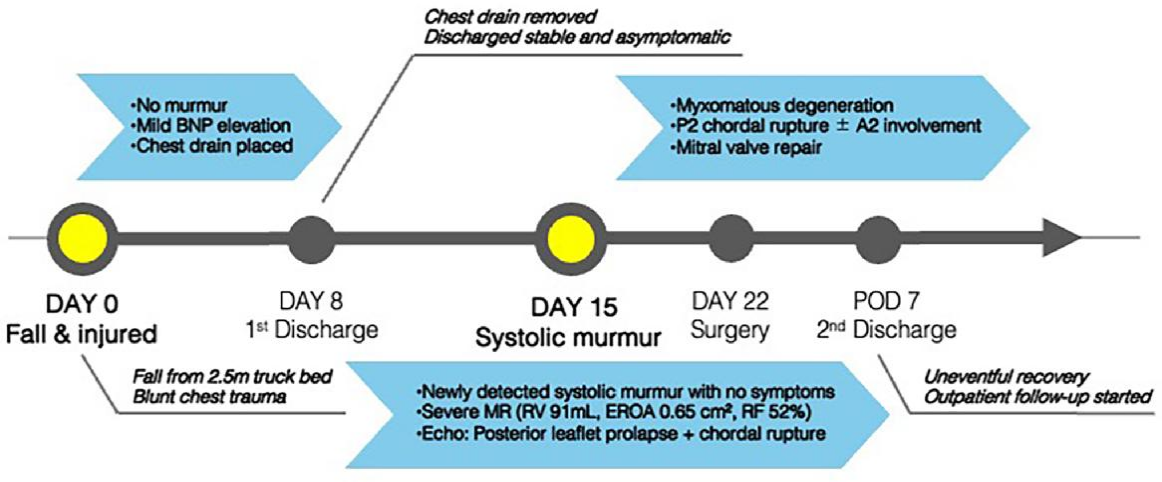


## Case presentation

**Figure 1:**
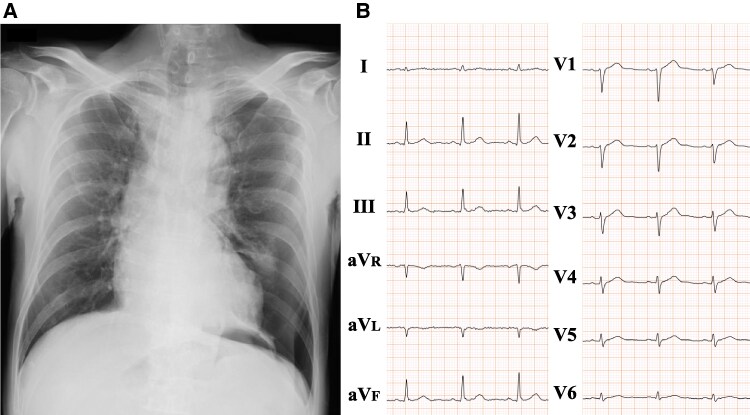
Chest radiography and electrocardiography of a 73-year-old man. Chest radiography confirmed a collapsed left lung parenchyma (*A*). Electrocardiography showed low voltage at the precordial leads (*B*).

**Figure 2:**
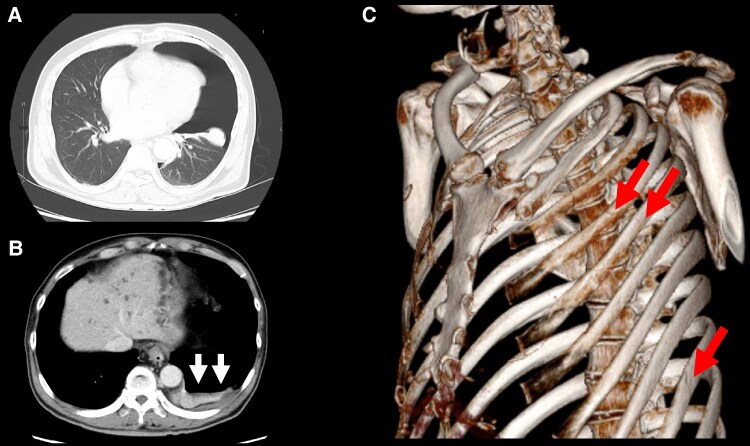
Chest computed tomographic images of a 73-year-old man. Chest computed tomography exhibited a collapsed left lung parenchyma (*A*) concomitant with blood components in the thoracic cavity (*B*, arrows), indicating haemopneumothorax. The three-dimensional image exhibited multiple rib fractures across the left fourth, fifth, and ninth ribs (*C*, arrows).

A 73-year-old man with a medical history of hypertension, dyslipidaemia, and hyperuricaemia was brought to our emergency department after falling ∼2.5 m from a truck bed, landing on his left side while working on his farm. Upon arrival, the patient was alert and haemodynamically stable, with a blood pressure of 156/117 mmHg, a heart rate of 92 b.p.m., and an oxygen saturation of 96% on ambient air. However, he was unable to speak or move independently due to severe trauma-related pain. Physical examination revealed marked tenderness around the left nipple and an abrasion on the left elbow. Chest auscultation revealed diminished breath sounds on the left side, although no audible heart murmur was detected at the time. Chest radiography showed a collapsed left lung (*Figure 1A*), and a 12-lead electrocardiogram indicated low voltage in the precordial leads, likely due to the pneumothorax (*Figure 1B*). Laboratory tests showed a slightly elevated brain natriuretic peptide (BNP) level of 34.0 pg/mL (normal value < 18.4 pg/mL). Chest computed tomography demonstrated a collapsed left lung (*Figure 2A*) with intrathoracic blood, indicating haemopneumothorax (*Figure 2B*). Additionally, multiple rib fractures were identified at the left fourth, fifth, and ninth ribs (*Figure 2C*). A chest drain was inserted, and conservative management was initiated for the rib fractures. After 7 days of observation and pain management, the patient was discharged on the eighth day without any medications, including diuretics.

**Figure 3:**
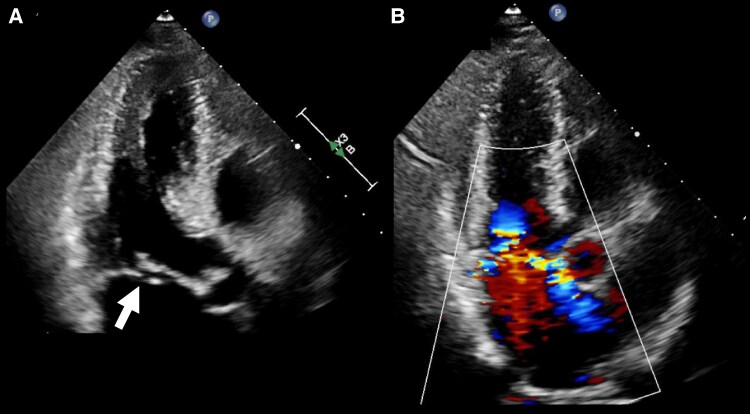
Two-dimensional transthoracic echocardiography of a 73-year-old man. Transthoracic echocardiography revealed prolapse of the posterior mitral leaflet with mobile filamentous structures attached to the leaflet tip (*A*, white arrow), suggesting chordal rupture. Severe eccentric regurgitant jet toward the anterior left atrial wall was observed (*B*). LA, left atrial.

**Figure 4:**
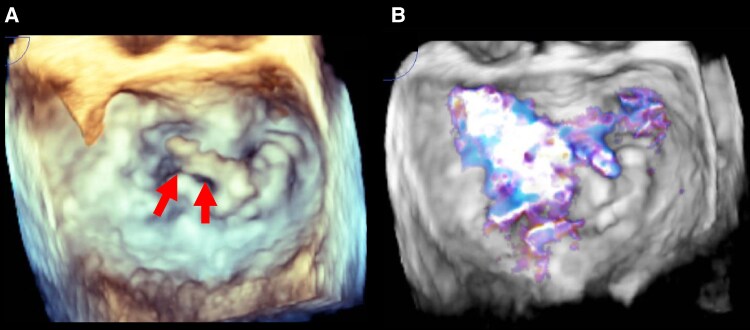
Three-dimensional transoesophageal echocardiography of a 73-year-old man. Transoesophageal echocardiography confirmed mitral leaflet prolapse resulting from chordal rupture of the middle scallop of the posterior mitral leaflet (*A*, arrows) concomitant with significant regurgitant jet (*B*).

**Figure 5:**
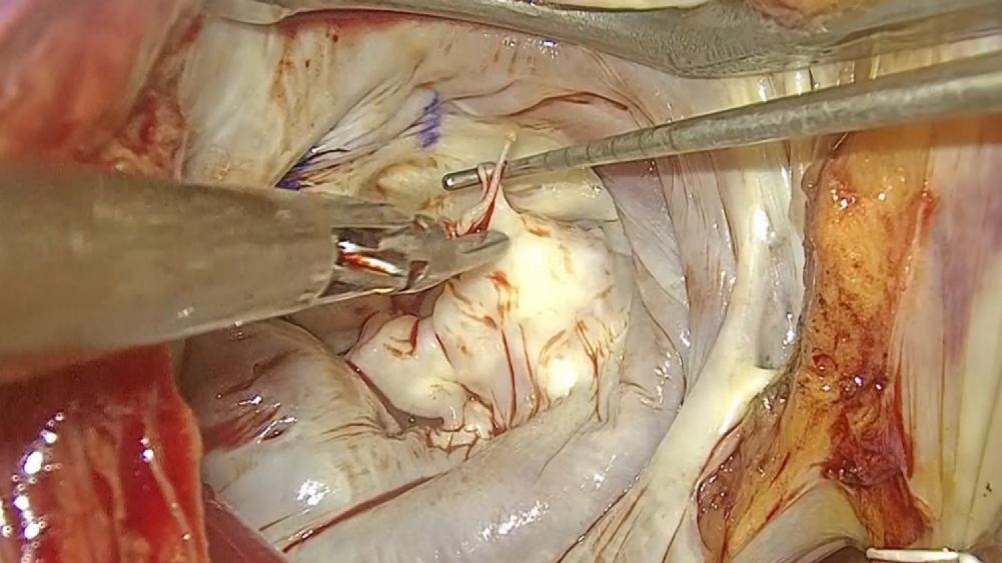
Intraoperative findings of the mitral valve of a 73-year-old man. Intraoperative inspection confirmed the presence of ruptured chordae mainly at the P2 segment, concomitant with moderate myxomatous degeneration.

One week later, however, he was referred back by his primary physician for evaluation of a newly detected heart murmur. Despite remaining asymptomatic, a Grade III systolic murmur was auscultated at the apex, radiating to the sternum. The BNP level was elevated to 58.0 pg/mL, compared with the level at the initial presentation. Transthoracic echocardiography showed posterior mitral leaflet prolapse with mobile filamentous structures at the leaflet tip, indicative of chordal rupture (*Figure 3A* and Supplementary material online, *Video S1*). Significant eccentric MR directed toward the anterior left atrial (LA) wall was observed, with a regurgitant volume of 64.8 mL and an effective regurgitant orifice area of 0.46 cm^2^ by volumetric method (*Figure 3B* and Supplementary material online, *Videos S2* and *S3*). On Doppler echocardiography, transmitral flow demonstrated a significantly high E-wave velocity of 117 cm/s with an E/A ratio of 1.2, and pulmonary venous flow analysis revealed a marked reduction of the S-wave velocity and the presence of systolic flow reversal into the pulmonary vein. These semiquantitative and qualitative Doppler findings were also consistent with severe mitral regurgitation. Although the pressure gradient of the tricuspid regurgitation was only 29 mmHg, the left ventricle (LV) demonstrated hyperdynamic wall motion, with an LV ejection fraction of 76% (see Supplementary material online, *Video S4*) and minimal LV and LA remodelling (LV end-diastolic volume of 119 mL; LA volume index of 44 mL/m^2^), indicating an acute origin. Preoperative transoesophageal echocardiography confirmed prolapse of the middle scallop of the posterior mitral leaflet due to chordal rupture (*Figure 4A* and *B*, Supplementary material online, *Videos S5* and *S6*). Intraoperative inspection confirmed rupture of the chordae primarily at the P2 segment, with mild myxomatous degeneration and partial involvement of the A2 segment (*Figure 5*). Mitral valve repair was successfully performed using Gore-Tex® CV4 sutures (three loops for the posterior and two for the anterior leaflet) with an annuloplasty ring. The postoperative course was uneventful, and the patient was discharged on postoperative Day 7. He continues to be followed on an outpatient basis.

## Discussion

Cardiac injury occurs in ∼9%–21% of patients with blunt chest trauma, potentially affecting the myocardium, pericardium, or coronary arteries, with the majority involving cardiac contusions.^8^ In contrast, traumatic valvular injury is extremely rare. Among the affected valves, the aortic valve is most commonly involved, followed by the tricuspid valve, while injury to the mitral valve is exceedingly rare.^1,2,9^ Consequently, cases of traumatic MR are primarily reported in isolated case reports.^3–8,10,11^

Mitral valve injury typically occurs during the vulnerable phases of the cardiac cycle—specifically isovolumetric contraction or end-diastole—when the LV is compressed between the sternum and spine by sudden external force.^6,11,12^ Experimental models suggest that intracardiac pressures exceeding 320 mmHg are required to rupture the mitral valve^13^; consequently, most cases occur in young individuals subjected to high-energy trauma, such as car crashes or motor vehicle accidents.^3,10^ In our case, however, the injury followed a relatively minor fall. It is plausible that preexisting senile myxomatous degeneration contributed to the patient’s susceptibility to chordal rupture, as confirmed intraoperatively. Hence, one can speculate that even lower-energy trauma may cause valve injury, especially in elderly patients, underscoring the need for heightened clinical suspicion.

Although acute MR typically presents with dramatic symptoms such as dyspnoea, pulmonary oedema, and heart failure, these may be masked in polytrauma settings by traumatic pain, pneumothorax-induced dyspnoea, altered mental status, or mechanical ventilation, all of which contribute to diagnostic delays. In the present case, the patient’s lack of subjective awareness of symptoms was likely attributable to the aforementioned factors. Additionally, the patient was largely sedentary after discharge, with markedly reduced levels of physical activity, which could have further obscured the clinical manifestations of heart failure. In this case, no cardiac murmur was initially auscultated, most likely due to the interposition of an air layer from the pneumothorax, which attenuated the transmission of cardiac sounds. The emergence of an audible murmur following thoracic drainage supports this hypothesis. Another plausible hypothesis for the delayed diagnosis is that the chordae may have sustained minor and partial initial trauma, which subsequently progressed to complete rupture over time.^4,5^ During this progressive clinical course, the severity of MR may have gradually increased—from mild to moderate and ultimately to severe—allowing the cardiovascular system sufficient time to adapt haemodynamically. While it remains speculative, this stepwise progression could explain the absence of acute symptoms and the delayed clinical recognition of the valvular injury.^4,5^ This hypothesis is further supported by the intraoperative finding of multiple ruptured chordae and the patient’s asymptomatic presentation despite the presence of subacute severe MR.

In patients with blunt chest trauma, clinical attention is often directed toward overt external injuries; however, the possibility of underlying cardiac complications must always be considered. It is reported that assessing cardiac biomarkers, particularly high-sensitivity troponin within 24 h post-trauma, can facilitate the diagnosis of traumatic cardiac injury and enable timely clinical intervention.^14^ Furthermore, all patients with blunt chest trauma—especially those exposed to moderate or greater impact, as in this case—should undergo a comprehensive physical examination and detailed echocardiographic evaluation to avoid missing potentially life-threatening cardiac injuries. In retrospect, a comprehensive echocardiographic examination should have been performed during the patient’s initial hospitalization. Although this oversight fortunately did not lead to any major clinical consequences, it is important to recognize that traumatic cardiac injuries may include myocardial contusion, valvular incompetence, coronary artery injury, haemopericardium, and even ventricular aneurysm formation. In the present case, had echocardiographic evaluation been undertaken at an appropriate time, the diagnosis of traumatic mitral regurgitation might have been established at an earlier stage. From a diagnostic perspective, while transthoracic echocardiography plays a pivotal role in identifying traumatic valvular dysfunction, its utility may be compromised by factors such as dressings over trauma sites, suboptimal patient positioning, limited acoustic windows, and artefacts associated with pneumothorax. When transthoracic imaging proves inconclusive—particularly in the presence of unexplained haemodynamic instability or newly detected murmurs—transoesophageal echocardiography should be promptly pursued.^4^

## Conclusions

Although traumatic mitral valve injury typically arises from forceful compression of the cardiac chambers between the sternum and vertebral column during sudden, high-impact trauma, this case illustrates that even less severe trauma can result in valvular injury—particularly in elderly individuals with preexisting degenerative valvular changes. Consequently, a high index of suspicion must be maintained to avoid overlooking this potentially life-threatening complication in cases of blunt chest trauma.

## Lead author biography


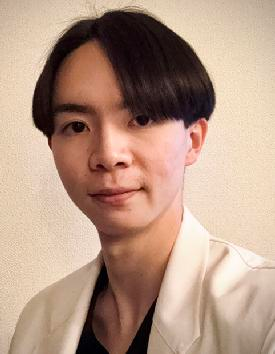


Shinnosuke Tsukamoto is a resident physician at Hyogo Prefectural Tamba Medical Centre, Japan. He has been specializing in cardiology since 2025.

## Supplementary Material

ytaf399_Supplementary_Data

## Data Availability

Data is accessible upon reasonable request; however, the authors reserve the right to individually assess and decide upon each request.
